# Digital twin framework for postural tachycardia syndrome and autonomic disorders

**DOI:** 10.3389/fneur.2025.1678955

**Published:** 2025-10-10

**Authors:** Peter Novak

**Affiliations:** ^1^Department of Neurology, Mass General Brigham, Boston, MA, United States; ^2^Harvard University, Boston, MA, United States

**Keywords:** postural tachycardia syndrome, autonomic disorders, digital twin, artificial intelligence, personalized medicine, orthostatic intolerance

## Abstract

Autonomic disorders, especially those characterized by orthostatic intolerance such as Postural Tachycardia Syndrome (POTS), remain diagnostically and therapeutically challenging due to their complex pathophysiology and limited access to specialized care. This paper proposes a conceptual framework for applying digital twin technology to POTS and other autonomic disorders. A digital autonomic twin—a dynamic, virtual replica of a patient’s autonomic system—offers a transformative approach to understanding, predicting, and managing these conditions. A dynamic digital twin framework integrates mechanistic and AI-based modeling utilizing continuous physiological, clinical, genetic, and patient-reported data to enhance individualized diagnosis, disease monitoring, and treatment. This system can simulate autonomic responses, predict disease trajectories, and personalize interventions. Digital twins provide real-time physiological modeling, adaptive treatment simulations, lifestyle intervention tracking, and integration of environmental and biometric data. Key components include wearable devices, electronic health records, AI-driven simulations, and clinician interfaces. However, challenges such as data volume, model transparency, and ethical considerations must be addressed. In conclusion, digital twin technology has the potential to revolutionize the management of POTS and related autonomic disorders, transitioning to personalized, predictive, adaptive medicine by providing a continuously updated and tailored approach to neurological care.

## Introduction

Autonomic disorders, particularly those related to orthostatic intolerance such as postural tachycardia syndrome (POTS), are common conditions affecting at least 3 million Americans (1, 2). These disorders are associated with orthostatic dizziness, fatigue, brain fog, insomnia, chronic pain and can be severely disabling (1, 3–5). Many of these conditions follow a preceding viral infection, including the SARS-CoV-2 virus (6).

POTS and other autonomic disorders pose significant diagnostic and treatment challenges due to their multifactorial nature, diverse manifestations, and lack of specialists and specialized autonomic testing laboratories (4, 7).

Key features of autonomic disorders are orthostatic intolerance associated with symptomatic cerebral hypoperfusion, which is accompanied by characteristic autonomic cardiovascular features (1, 8). For example, POTS is defined as combination of chronic orthostatic intolerance symptoms and orthostatic tachycardia (1). Because autonomic disorders produce quantifiable physiological alterations (such as heart rate, blood pressure and cerebral blood flow changes), they are particularly well-suited to a digital twin (DT) approach. This offers a promising pathway for individualized diagnosis, monitoring, and treatment optimization.

### Digital twin framework

Digital twins were developed to create virtual replicas of physical systems (9–11). Their adoption in healthcare is rapidly expanding (12–14) with applications in cardiology (10, 15), neurology (16), metabolic disorders (17), treatment of sepsis (18) and other areas.

A digital twin can be defined as an interconnected system that integrates simulations, real-time sensor data, visualization tools, and interactions between physical and digital counterparts (9). Its distinguishing characteristic is adaptability: it continuously learns from historical data, assimilates information from external sources, and performs virtual experiments to enhance predictive accuracy and system behavior over time. The principal distinction between a conventional simulation and a digital twin lies in the latter’s integrative and adaptive nature. Whereas simulations are typically isolated and designed for specific scenarios, digital twins are dynamic, continuously evolving models that reflect and respond to changes in the real-world system they represent.

**Figure 1:**
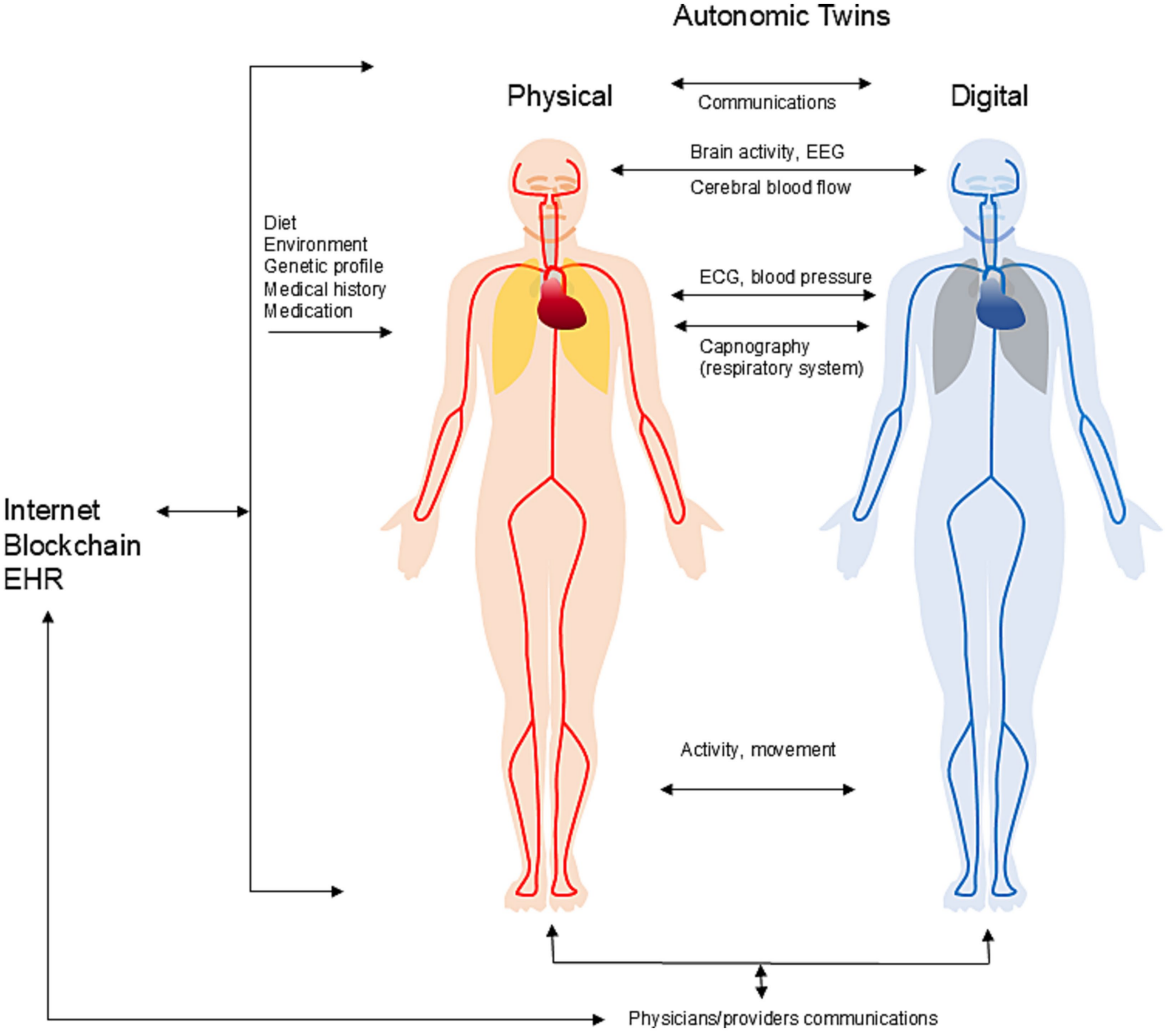
The concept of digital twins for autonomic disorders.

Designing a digital twin for POTS and for autonomic disorders in general involves creating a virtual, personalized model of a patient’s autonomic cardiovascular system, utilizing real-time data to assess disease status, predict disease progression, and optimize treatment (Figure 1). This framework integrates clinical data, genetic information, patient characteristics, and continuous sensor data to mimic the patient’s physiological systems and organs.

## Methodology

DT relies on interdisciplinary collaboration. The development and implementation of digital twins for autonomic disorders require close collaboration between clinicians, researchers, data scientists, and engineers. Collaboration between AI developers and clinicians is essential. The digital twin framework is a dynamic, continuously evolving adaptive system.

### System architecture

The digital twin consists of several subsystems, including:

1. Monitoring: Data acquisition using multiple data streams.
2. Modeling: Incorporating mechanistic and artificial intelligence (AI) models of autonomic function.
3. Simulations: Running simulations of disease and treatment scenarios.
4. Patient-Physician interaction: Facilitating communications between the patient, digital twin and clinician.

### Data acquisition

The digital twin is built and maintained by continuously collecting patient data from a variety of sources, including:

*Wearables*: Devices that monitor vital signs like heart rate, blood pressure, end-tidal CO_2_, electroencephalogram cerebral blood flow and other signals in real time.
*Electronic health records*: Historical data, including previous diagnoses, medical history, lab results, medication, and more.
*Patient-reported outcomes*: Self-reported data on symptoms, activity levels, sleep patterns, stress levels, and medication adherence.
*Environmental factors*: Contextual data, such as weather conditions, may influence POTS symptoms.
Handling sensitive patient data, especially in real-time, would require stringent data privacy protocols.

AI-driven digital twin modeling includes integrating machine learning, deep learning, and AI algorithms with simulation tools to create more dynamic, data-driven, and predictive models. Several publicly available, mostly open-source, Python-based platforms are available for physical systems, including Simply (19), Open Digital Twin Project (20) and PyTwin (21). TumorTwin software developed for cancer patients, is also available (22).

## Discussion

The concept of digital twins of POTS and autonomic disorders is a new paradigm with the potential of a transformative approach to understanding, predicting, and managing these conditions.

Originally, digital twins were defined as virtual replicas of physical systems (9). In autonomic medicine, where comprehensive mechanistic models are still in early stages, the digital twin approach may rely primarily on AI-based modeling. Nonetheless, mechanistic models may still be applied for specific physiologic domains, such as simulating heart rate responses to orthostatic stress or the effect of hyperventilation on cerebral blood flow. While AI models offer powerful predictive capabilities, they can lack transparency and often function as “black-boxes” (18). Ensuring model transparency is essential so that both clinicians and patients can understand how decisions and recommendations are made. Conducting simulations across a range of outcomes and interventions can help improve the transparency of AI-driven models.

AI-powered digital twins can also analyze vast amounts of research, clinical trials, and patient data to build evolving models that predict how a patient’s condition might progress. This empowers clinical teams to test hypotheses and develop new treatment plans more rapidly, potentially speeding advances in both research and care. Furthermore, digital twin is adaptive, updating in real time as new data arrives. Even with incomplete or imprecise information, these models improve progressively, learning from the patient’s unique responses to treatments, activities, or lifestyle changes.

A significant challenge will be the need to process and store vast amount of data. While some data such as genetic footprint for an individual patient should be stored indefinitely, for real-time physiologic data some form of compression or filtering will be necessary. Decentralized blockchain technology (23) can be advantageously applied to digital twin health care data.

Digital twins may help bridge the gap between basic and clinical research. Basic research focuses on physiological and molecular disease mechanisms, while clinical research addresses treatment efficacy and patient outcomes. This division often delays translating discoveries into practice. Digital twins allow immediate integration of basic research insights—such as autonomic dysfunction or blood volume regulation—into patient-specific models. This integration enables real-time predictions, deeper understanding, and faster testing of therapies.

Digital twins unify structural and functional patient data into comprehensive models. By combining imaging, lab tests, and wearable sensor data, they account for anatomical changes and physiological dysfunctions together. This approach may explain how functional symptoms (e.g., tachycardia on standing) may arise from subtle structural issues. It moves beyond the traditional debate between structural versus functional disorders, offering an integrated perspective and enabling more precise treatments targeting both aspects.

**Figure 2:**
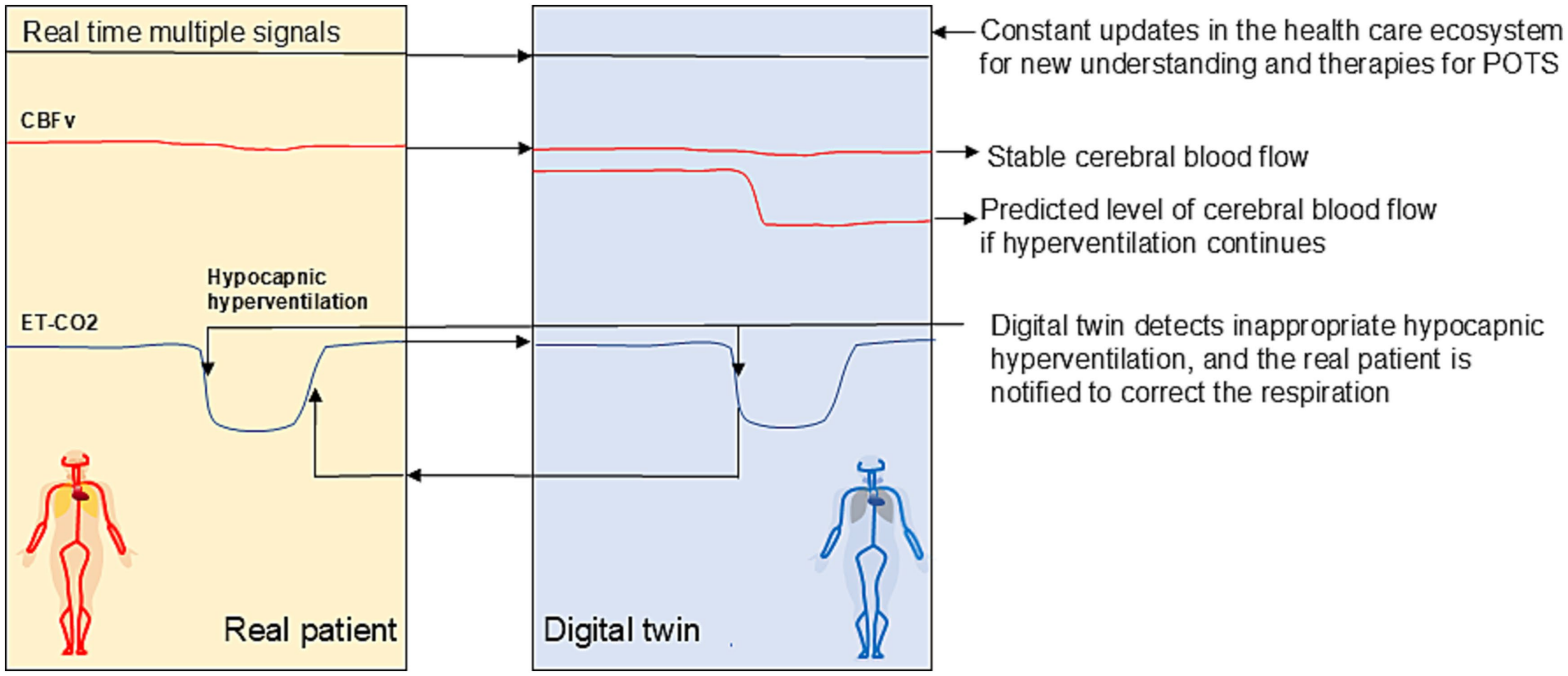
A digital twin-based system for early detection and prevention of cerebral hypoperfusion in POTS. Orthostatic lightheadedness due to cerebral hypoperfusion is common and one of the most disabling symptoms of POTS and is due to hypocapnia-induced cerebral arteriolar vasoconstriction (8). The digital twin continuously monitors physiological signals such as respiration rate, blood pressure, and metabolic state and evaluates metabolic demand. In the event of inappropriate hyperventilation (e.g., not due to exercise or metabolic demand), the system identifies it as a precursor to cerebral vasoconstriction and reduced blood flow, which can lead to dizziness. The digital twin alerts the patient to slow their breathing, first through subconscious vibration signals, then with auditory cues, and, if necessary, through an escalation to the patient’s physician for further investigation. Hyperventilation is a good proxy for cerebral hypoperfusion, since its causes cerebral arteriolar vasoconstriction and is a common cause of dizziness in postural tachycardia syndrome (POTS). It often occurs a few seconds before measurable hypoperfusion and dizziness allowing to act preemptively, giving a window of time to intervene before symptoms occur. Direct cerebral blood flow measurements are simultaneously conducted to verify the effect of hyperventilation on perfusion. This approach may potentially prevent symptoms like dizziness before they occur. CBFv = cerebral blood flow, ET-CO_2_ = end tidal CO_2_.

Clinicians would interact with digital twins through intuitive dashboards integrated with electronic health records. For instance, when treating a POTS patient, a doctor could review the digital twin’s current status—heart rate, blood pressure, autonomic function—and receive suggested interventions (Figure 2). As such digital twin framework expands the concept of telemedicine. Future iterations might even enable real-time virtual consultations between the patient’s digital twin and a “virtual” physician, using verbal and non-verbal communication. They may also allow virtual clinical trials, where therapeutic efficacy is first tested within the DT before real-world application.

Digital twins enable real-time simulation of patient-specific medication responses, helping predict which drugs will be most effective. For example, POTS patients often respond differently to medications like pro-amatine or pyridostigmine due to their unique autonomic profiles (1, 5). A digital twin can guide personalized dosing, reducing trial-and-error and minimizing side effects.

AI can also forecast potential complications such cardiovascular instability, allowing clinicians to intervene preventatively rather than reactively. By continuously monitoring data from wearables or other devices, digital twins can detect subtle warning signs—like changes in heart rate or blood pressure variability —that precede acute episodes, thereby reducing complications of disease, hospitalizations and improving outcomes.

Because digital twins are continuously updated, and the real patient – digital twin connection is in real time, they can provide immediate feedback on how lifestyle changes affect a patient’s health. For example, if a patient with POTS begins an exercise program (24), the digital twin could monitor vital signs like heart rate, blood pressure and respiratory pattern in real time, allowing adjustments to the plan as needed.

Digital twins can enhance lifestyle interventions for POTS by integrating real-time data and offering personalized recommendations. They can simulate patient responses to various activity levels—predicting tolerance to increased exercise or standing durations—and suggest optimal regimens.

Dietary management is another key area, as POTS symptoms often respond to changes in salt intake and hydration (4). Digital twins can monitor biometrics in relation to diet, adjusting nutritional advice accordingly and tracking related metrics like weight or sodium levels.

Combined with wearable sleep trackers, digital twins can also assess sleep quality and tailor recommendations, addressing how poor sleep exacerbates symptoms and guiding personalized sleep hygiene strategies.

Overall, digital twins represent a revolutionary, patient-centered approach for managing POTS and related autonomic disorders. By simulating individualized disease progression and treatment responses, they bridge the gap between reactive and proactive care. At a broader level, digital twins could serve as a frontline intervention tool—if effective in simulation, therapies could then be applied to the real patient.

With continued development, validation, and ethical oversight, digital twins may soon become an integral part of routine clinical care.

## Conclusion

Digital twins represent a transformative, patient-centric approach to managing POTS and other autonomic disorders. By integrating real-time data, physiological modeling, and predictive analytics, DTs enable a shift from reactive to proactive, personalized neurological care. With appropriate validation and ethical implementation, they hold the potential to become a cornerstone of future clinical practice in autonomic neurology.

## Data Availability

The original contributions presented in the study are included in the article/supplementary material, further inquiries can be directed to the corresponding author/s.
